# Zoledronic acid and denosumab are associated with similar fracture incidence and mortality in patients with type 2 diabetes: a population-based cohort study

**DOI:** 10.3389/fendo.2025.1590472

**Published:** 2025-08-04

**Authors:** Vanessa Rouach, Hilary Gortler, Yona Greenman, Gabriel Chodick, Inbal Goldshtein

**Affiliations:** ^1^ Institute of Endocrinology, Diabetes, Hypertension and Metabolism, Sourasky Medical Center, Tel Aviv, Israel; ^2^ Epidemiology Department, School of Public Health, Gray School of Medecine, Tel Aviv University, Tel Aviv, Israel; ^3^ Sackler School of Medicine, Tel Aviv University, Tel Aviv, Israel; ^4^ Maccabitech Institute for Research and Innovation, Maccabi Healthcare Services, Tel Aviv, Israel

**Keywords:** anti-resorptive treatment, type 2 diabetes, osteoporosis, fracture prevention, mortality

## Abstract

**Purpose:**

To assess the comparative effectiveness of zoledronic acid *vs*. denosumab in prevention of major osteoporotic fractures and mortality among patients with type 2 diabetes.

**Methods:**

The study population was identified by crosslinking the diabetes and osteoporosis registries of a large healthcare organization in Israel. Demographics, Charlson Comorbidity Index (CCI), diabetes complications, bone mineral density (BMD) T-scores, hemoglobin A1c levels, eGFR, purchase of statins and anti-resorptive agents were collected. Exposure groups were matched using propensity score. Kaplan-Meier curves were generated to assess the time from treatment initiation to outcomes. Multivariable Cox’s proportional hazards survival models estimated hazard ratios (HR) and 95% CIs for each outcome.

**Results:**

Among 27503 patients with concurrent osteoporosis and type 2 diabetes, 627 (4.7%) received zoledronic acid and 502 (3.7%) denosumab. Prior to matching, the denosumab-treated patients were older (mean age 75.7 *vs* 71.9, p<0.01), had longer diabetes duration (mean 8.4 *vs* 7.2 years, p<0.01), and had a lower baseline eGFR (59.4 *vs* 75.3, p<0.01) than the zoledronic acid-treated patients. After matching, 415 pairs of subjects were included. The incidence of all examined outcomes was similar in the Zol and Dmab treatment groups, including 5-year cumulative incidence of fractures (38% *vs* 31%), death events (36% *vs* 41%), overall fracture risk (HR=1.17, 95% CI: 0.78 to 1.75), death risk (HR= 1.12, 95% CI:0.87 to 1.44), and mortality after a hip fracture (HR= 0.92, 95% CI:0.37-2.29).

**Conclusions:**

Our findings suggest comparability of Zoledronic Acid and Denosumab in managing osteoporotic fractures and mortality among patients with type 2 diabetes.

## 1 Introduction

As the global population ages, osteoporosis is becoming an increasingly significant burden on healthcare systems (1–3). Concurrently, diabetes mellitus contributes substantially to morbidity and mortality in older adults and has been identified as a factor that exacerbates bone fragility in individuals with osteoporosis (4–7). Notably, patients with type 2 diabetes face an elevated risk of fractures at various skeletal sites, despite often presenting with normal bone mineral density (BMD) and low bone turnover rates. These observations have raised concerns about the efficacy of anti-resorptive therapies in this population (8–11).

To date, evidence regarding the efficacy and safety of anti-resorptive agents in individuals with type 2 diabetes is primarily derived from observational studies and *post-hoc* analyses of randomized controlled trials (RCTs) (12–17), as no RCTs have been specifically conducted in this patient group. Moreover, only a limited number of studies have directly compared the safety and effectiveness of different anti-resorptive agents. A prior meta-analysis of nine RCTs reported that denosumab and bisphosphonates demonstrated similar efficacy and safety in reducing fracture risk, although denosumab was associated with greater improvements in BMD (18). In a population-based cohort study, Choi et al. found that denosumab and zoledronic acid had comparable safety and effectiveness regarding the risk of serious infections, cardiovascular disease, and osteoporotic fractures in a relatively young cohort (19). A study by Curtis et al. presented at the American Society of Bone And Mineral Research annual convention in 2023 found denosumab to be more effective than zoledronic acid in preventing major osteoporotic fractures among post-menopausal women with osteoporosis (20). However, a study by Alarkawi et al. suggested a higher mortality risk associated with denosumab compared to oral bisphosphonates (21), and unlike bisphosphonates, the impact of denosumab on mortality remains underexplored (22–24).

While bisphosphonates remain a cornerstone in the treatment of osteoporosis, oral formulations can present adherence challenges and gastrointestinal side effects (25, 26), particularly in elderly patients with multiple comorbidities. Zoledronic acid, a potent intravenous bisphosphonate with extended dosing interval and distinct pharmacological profile, and denosumab, a subcutaneous RANKL inhibitor, offer non-oral alternatives with proven efficacy in fracture prevention (27–29). However, direct comparative data on the long-term safety and effectiveness of these two agents remain limited, especially in high-risk populations such as individuals with type 2 diabetes. Further evidence is needed to inform optimal therapeutic strategies in this growing and vulnerable group. Therefore, the aim of this study was to compare the long-term risk of major osteoporotic fractures and all-cause mortality among patients with type 2 diabetes treated with either zoledronic acid or denosumab—two widely prescribed, potent, non-oral anti-resorptive agents.

## 2 Subjects and methods

The study utilized longitudinal data from Maccabi Healthcare Services (MHS). MHS is the second largest healthcare provider and insurer in Israel, covering over two million members, which accounts for approximately 25% of the population with a countrywide distribution. The insured population is nationally representative because according to the 1995 national health insurance law, health medical organizations (HMOs) may not deny coverage to applicants on any grounds, including age or state of health. MHS’s central database contains patient demographics, diagnoses, medical procedures, hospitalizations, and full capture of all prescription medication dispensations and laboratory tests since 1999. MHS has developed several computerized registries of major chronic diseases, such as oncologic diseases, diabetes, and osteoporosis that are continuously updated. The study follow-up period extended from January 1998 to December 2021.

The current study obtained data from the MHS diabetes and osteoporosis registries. Registry assembly has been previously described elsewhere, and a comprehensive approach was used to cross-validate them and ensure high specificity (30–32).

Briefly, the osteoporosis registry identifies patients by diagnoses, by at least two dispensations of medications for osteoporosis, by bone mineral density (BMD) in the osteoporotic range (*T*-score ≤ − 2.5), or by a major osteoporotic fracture (MOF) which occurred at a typical age (50+ years for females and 60+ years for males).Registry entry date is the earliest of all the above criteria.

Major osteoporotic fractures (MOF) were identified using ICD-9/ICD-10 diagnostic codes recorded in the Maccabi Healthcare Services electronic medical records, clinician diagnoses and records of fracture-related procedures. MOF sites included the hip, spine, distal radius and proximal humerus fractures, in accordance with fracture risk assessment definitions (33). Only low-trauma fractures, excluding those related to high-energy trauma or pathological fractures, were included. The diagnosis codes were verified using internal registry validation processes previously described in MHS epidemiological research (27).

The diabetes registry identifies patients according to HbA1c values and glucose test results, DM therapy dispensations, and a diagnosis of DM from relevant physicians, with an overall specificity of 99.99%. Considering the different pathogeneses of type 1 and type 2 diabetes, the current study focused on type 2 diabetes compared to no diabetes.

### 2.1 Participants

The study population was identified through cross-linkage of the Maccabi Healthcare Services (MHS) diabetes registry and osteoporosis registry. Eligible participants were men and women aged over 50 years who had been entered into the diabetes registry prior to their osteoporosis diagnosis. The index date was defined as the initiation of treatment with either denosumab (Dmab) or zoledronic acid (Zol). As treatment duration was not consistently recorded and could not be reliably assessed, the analysis was based on treatment initiation rather than cumulative exposure. Patients receiving other osteoporosis treatments or with a history of cancer, multiple myeloma, or Paget’s disease of bone were excluded. As previously described, inclusion in the osteoporosis registry was based on documentation of bone mineral density (BMD) in the osteoporotic range, a major osteoporotic fracture (MOF), or initiation of anti-osteoporotic therapy. None of the included participants had a history of prior fractures or previous anti-osteoporotic treatment.

### 2.2 Outcomes

The primary outcome was MOF (hip, spine, distal radius and proximal humerus fractures).

As previously noted, 44% of patients were enrolled in the osteoporosis (OP) registry following a fracture. The registry does not reliably distinguish between a new fracture event and a referral or follow-up related to a previous fracture, due to limitations in how clinician diagnosis files are recorded. To avoid misclassification bias, we limited this analysis to patients without any fracture diagnosis at baseline.

The secondary outcome was death.

### 2.3 Additional covariates

We extracted data on demographic and clinical variables, including age, body mass index (BMI), hemoglobin A1c levels, diabetes duration, presence of microvascular and macrovascular complications, insulin use, history of hypoglycemic events, falls, estimated glomerular filtration rate (eGFR), Charlson Comorbidity Index (CCI), and the purchase of statins and glucose-lowering agents. Additional variables included bone mineral density (BMD) T-scores, smoking history, alcohol consumption, diagnoses of rheumatoid arthritis, and chronic glucocorticoid use. Most variables were captured at the time of entry into both the diabetes and osteoporosis registries; for the purposes of this analysis, we used the values recorded at the time of osteoporosis registry entry.

### 2.4 Statistical analysis

Population demographic data were expressed as means with corresponding standard deviations and percentages for continuous and categorical variables, respectively. Cumulative incidence rates were calculated using 1 minus the Kaplan-Meier survival estimates. For the fracture outcome, censoring occurred due to death, HMO exit, or end of follow-up. For the mortality outcome, patients were censored at HMO exit or end of follow-up. Propensity score matching was performed to balance all covariates that showed a standardized mean difference (SMD) greater than 0.1. The propensity model was calculated via logistic regression and included: age, eGFR category, A1C levels, diabetes duration and smoking. Initial descriptive statistics were conducted in SPSS (IBM Corp. Released 2020. IBM SPSS Statistics for Windows, Version 27.0.). Matching, detailed descriptive statistics, cox regression and graphs were generated using R statistical software (version 4.3, R Core Team, 2022. Cox’s proportional hazards models were used to estimate adjusted hazard ratios (HR) and 95% CIs for each outcome. The models were adjusted for age and estimated glomerular filtration rate (eGFR). The proportionality assumption was assessed graphically and using Schoenfeld residuals. The analysis included all eligible patients (rather than a sample). The size of the matched population was sufficiently powered to detect HR’s of 1.2 or more assuming an outcome incidence of 40% and no drop-out, with a power of 80% and type 1 error of 5%. However, due to administrative censoring of 50% within 5 years, the detectable HR inflates to 1.34.

## 3 Results

A total of 27,503 patients with concurrent osteoporosis and type 2 diabetes were identified; 67% were women. The mean interval from diabetes diagnosis to osteoporosis diagnosis was 6.8 ± 5.7 years. The mean age at entry into the osteoporosis registry was 72.6 ± 8.6 years, with a mean diabetes duration of 6.8 ± 5.4 years. The mean body mass index (BMI) was 30.0 ± 5.4 kg/m², and the mean hemoglobin A1c (HbA1c) level was 6.7 ± 0.8%. Approximately 9% of the cohort had an HbA1c level >8%.

Mean bone mineral density (BMD) T-scores were −1.0 ± 0.3 at the total hip and −1.7 ± 1.2 at the femoral neck. Overall, 44% of patients were included in the osteoporosis registry following a fracture: 13.6% had sustained a hip fracture, 8.2% a vertebral fracture, and 22.2% non-hip, non-vertebral fractures. The mean age at the time of fracture was 74 ± 9 years.

Among the 1,129 patients treated with zoledronic acid or denosumab, 51.7% initiated treatment following a fracture: 21.0% after a hip fracture, 13.2% after a vertebral fracture, and 16.9% after a non-hip, non-vertebral fracture (including the humerus, radius, ribs, pelvis, or ankle). The mean follow-up time from treatment initiation was 3.5 ± 2.6 years in the zoledronic acid group and 3.4 ± 2.5 years in the denosumab group (*p* = 0.48), indicating comparable follow-up duration between the two treatment groups.

Prior to propensity score matching, patients treated with denosumab were significantly older than those treated with zoledronic acid (mean age 78 *vs*. 73 years, *p* < 0.01) and had a longer duration of diabetes (8.4 *vs*. 7.3 years, *p* < 0.01). Denosumab-treated patients were also more likely to be on insulin therapy (29.7% *vs*. 23.9%, *p* = 0.02) and had lower estimated glomerular filtration rates (eGFR; 59 *vs*. 75 mL/min/1.73m², *p* < 0.01). No significant differences were observed between the two groups in sex distribution, BMI, Charlson Comorbidity Index (CCI), smoking status, alcohol consumption, hip BMD T-score, HbA1c levels, microvascular complications, hypoglycemic events, or statin use. Following propensity score matching, 415 matched pairs of patients treated with zoledronic acid or denosumab were included in the final analysis (**Table 1**).

**Table 1:** Patients’ characteristics before and after matching.

Characteristic	Zol (N=627)	Dmab (N=502)	SMD	Zol (N=415)	Dmab (N=415)	SMD
Gender, n (%)			0.064			0.049
– Female	382 (61%)	290 (58%)		250 (60%)	240 (58%)	
– Male	245 (39%)	212 (42%)		165 (40%)	175 (42%)	
Age at index, Mean (SD)	73 (± 8)	78 (± 8)	0.59	76 (± 7)	76 (± 8)	0.015
BMI, Mean (SD)	29.9 (± 5.5)	29.6 (± 5.1)	0.060	30 (± 5.3)	29.5 (± 5.1)	0.10
CVD event, n (%)	239 (38%)	234 (47%)	0.17	185 (45%)	175 (42%)	0.049
Stroke event, n (%)	109 (17%)	88 (18%)	0.004	80 (19%)	72 (17%)	0.050
HbA1c, Mean (SD)	6.60 (± 0.71)	6.66 (± 0.78)	0.083	6.59 (± 0.71)	6.66 (± 0.80)	0.085
eGFR, Mean (SD)	75 (± 18)	59 (± 25)	0.74	70 (± 18)	70 (± 21)	0.023
eGFR category, n (%)			0.42			0.050
– (60+)	114 (18%)	73 (15%)		64 (15%)	67 (16%)	
– (0–30]	0 (0%)	22 (4.4%)		0 (0%)	0 (0%)	
– (30–60]	35 (5.6%)	67 (13%)		34 (8.2%)	39 (9.4%)	
– Missing	478 (76%)	340 (68%)		252 (63%)	252 (63%)	
Statin users, n (%)	216 (34%)	181 (36%)	0.034	143 (34%)	136 (33%)	0.036
Diabetes duration, Mean (SD)	7.3 (± 5.3)	8.4 (± 6.2)	0.2	7.7 (± 5.5)	8.1 (± 8.1)	0.10
Microvascular event, n (%)	384 (61%)	334 (67%)	0.11	270 (65%)	273 (66%)	0.015
CCI Mean (SD)	4 (± 3)	4 (± 3)	0.059	4 (± 3)	3 (± 3)	0.11
Smoking, n (%)			0.031			0.13
– Current smoker	22 (3.5%)	16 (3.2%)		14 (3.4%)	12 (3.0%)	
– Never	239 (38%)	188 (37%)		157 (38%)	133 (32%)	
– Past smoker	6 (1%)	4 (0.8%)		3 (0.7%)	3 (0.7%)	
– Unknown	360 (57%)	294 (59%)		241 (58%)	267 (64%)	

**Figure 1:**
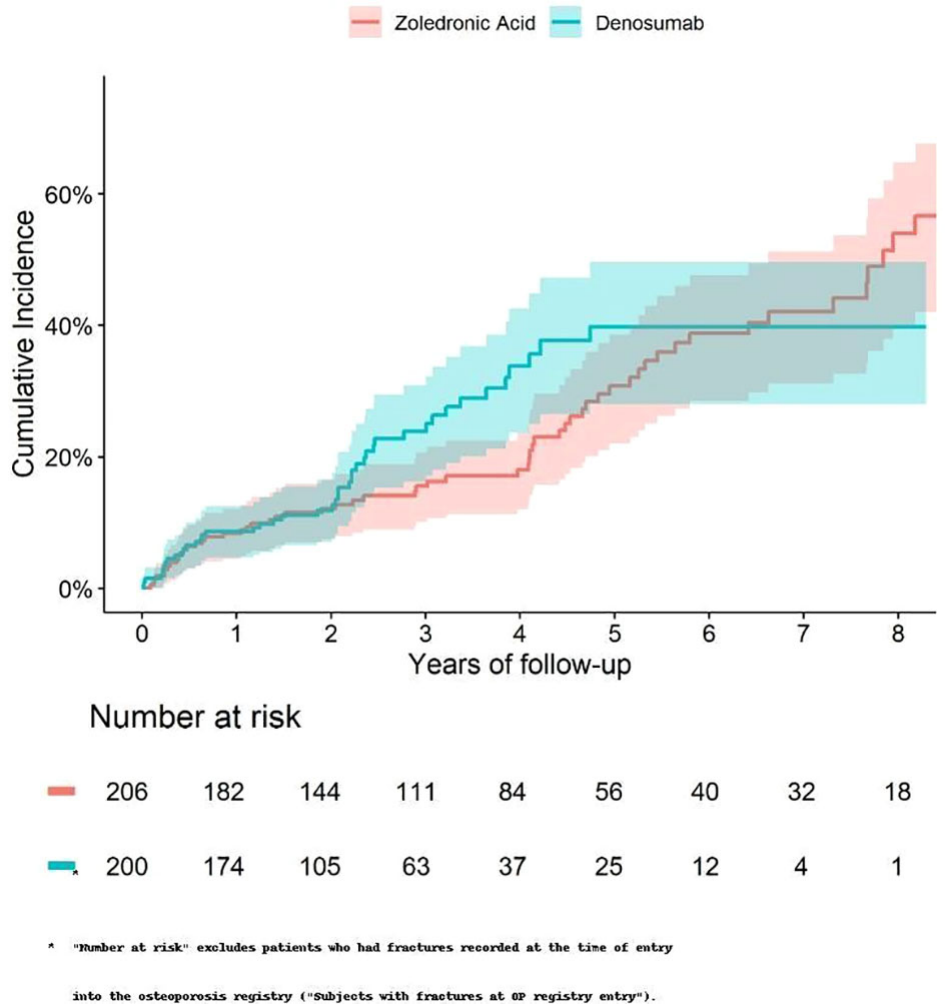
Cumulative incidence of fractures among fracture-free patients at OP registry entry, treated with Zol. *vs* Dmab.

**Figure 2:**
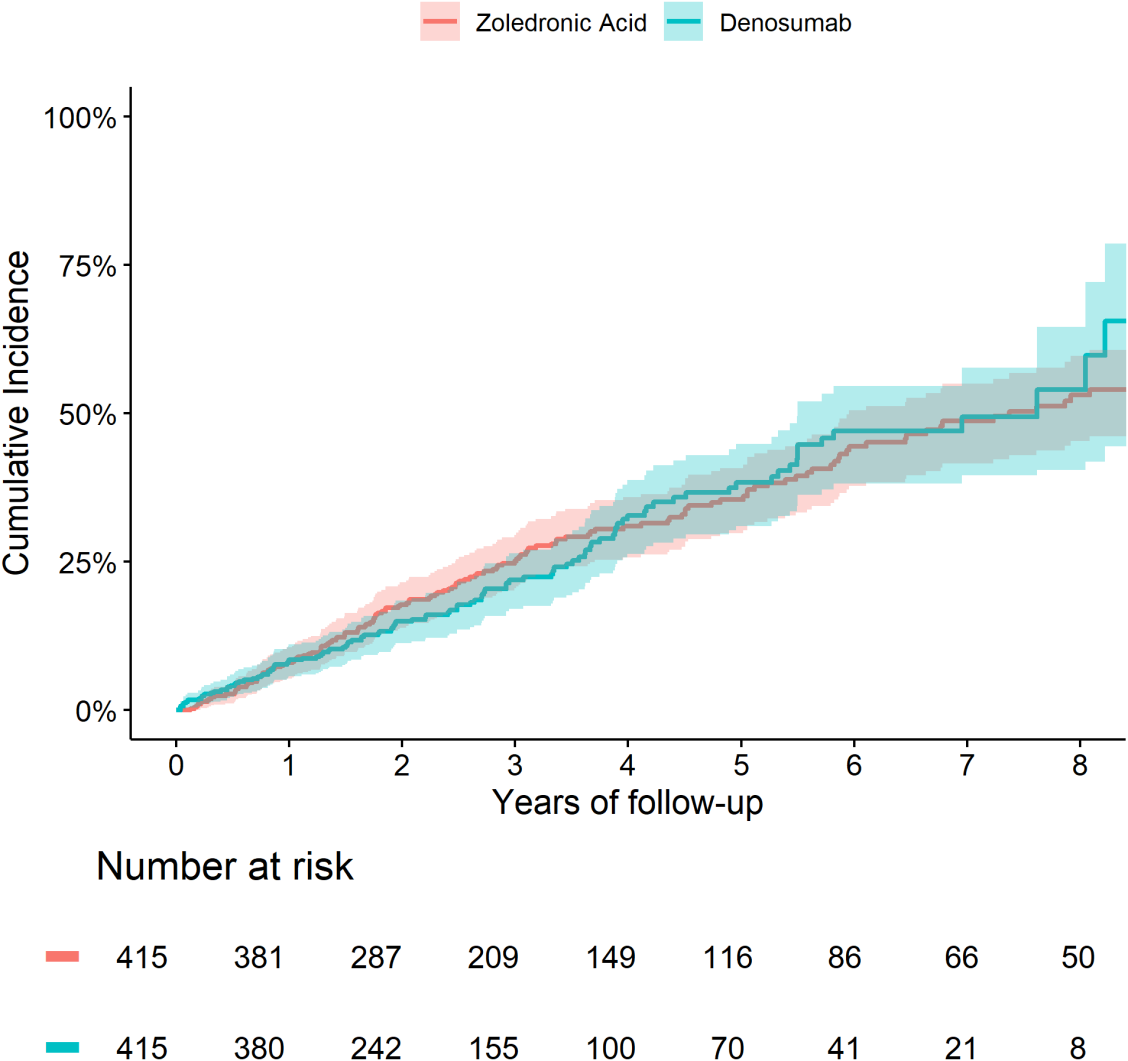
Cumulative incidence of death in patients treated with Zol. *vs* Dmab.

The 5-year cumulative incidence of fracture (**Figure 1**) and death (**Figure 2**) were similar in the Zol and Dmab treatment groups (38% *vs* 31%, and 36% *vs* 41% respectively). We did not observe any significant differences in the risk of fracture HR=1.17 (95% CI: 0.78-1.75) or death HR= 1.12 (95% CI: 0.87-1.44) between the two treatment groups.

We conducted additional analyses in two high-risk subgroups: patients aged over 75 years and those with a prior hip fracture.

In the older subgroup, after propensity matching, two pairs of 217 patients remained for analysis. Their characteristics are summarized in **Table 3**. The mean age was 81.6 ± 4.3 in Zol-treated patients and 82.0 ± 4.6 in Dmab-treated patients, mean estimated glomerular filtration rates were 66 ± 18 and 64 ± 20, mean HbA1C were 6.56 ± 0.67 and 6.68 ± 0.76 and the microvascular complication rates were 62% and 67% respectively (**Table 2**).

**Table 2:** Baseline characteristics of patients aged >75 years, derived from the matched cohort, pre and post-matching.

Characteristic	Zol (N=245)	Dmab (N=316)	SMD	Zol (N=217)	Dmab (N=217)	SMD
Gender, n (%)			0.07			0.05
– Female	137 (56.0%)	187 (59.2%)		122 (56.2%)	127 (58.5%)	
– Male	108 (44.0%)	129 (40.8%)		95 (43.8%)	90 (41.5%)	
Age at treatment, mean ± SD	81.2 ± 4.3	83.0 ± 5.1	0.37	81.6 ± 4.3	82.0 ± 4.6	0.10
BMI, mean ± SD	29.6 ± 5.2	29.7 ± 5.2	0.03	29.7 ± 5.2	29.6 ± 5.0	0.02
Cardiovascular disease, n (%)	122 (49.8%)	169 (53.5%)	0.07	107 (49.3%)	107 (49.3%)	<0.01
Stroke, n (%)	56 (22.9%)	60 (19.0%)	0.10	49 (22.6%)	42 (19.4%)	0.08
HbA1c, mean ± SD	6.61 ± 0.69	6.68 ± 0.73	0.11	6.56 ± 0.67	6.68 ± 0.76	0.17
Alcohol use, n (%)	1 (0.4%)	1 (0.3%)	<0.01	1 (0.5%)	1 (0.5%)	<0.01
eGFR category, n (%)			0.41			0.02
– (0–30]	0 (0.0%)	16 (5.1%)		–	–	
– (30–60]	22 (9.0%)	51 (16.1%)		22 (10.1%)	23 (10.6%)	
– (60+)	34 (13.9%)	41 (13.0%)		29 (13.4%)	30 (13.8%)	
– Missing	189 (77.1%)	208 (65.8%)		166 (76.5%)	164 (75.6%)	
eGFR, mean ± SD	67 ± 17	55 ± 24	0.57	66 ± 18	64 ± 20	0.11
Diabetes duration, mean ± SD	8.3 ± 5.7	9.3 ± 6.4	0.16	8.1 ± 5.6	9.3 ± 6.5	0.19
Microvascular event, n (%)	155 (63.3%)	212 (67.1%)	0.08	135 (62.2%)	145 (66.8%)	0.10
CCI, mean ± SD	4.0 ± 3.0	4.0 ± 3.0	0.06	4.0 ± 3.0	4.0 ± 3.0	0.05
Smoking, n (%)			0.06			0.16
– Current smoker	8 (3.3%)	8 (2.5%)		6 (2.8%)	5 (2.3%)	
– Never	92 (37.6%)	125 (39.6%)		80 (36.9%)	68 (31.3%)	
– Past smoker	1 (0.4%)	1 (0.3%)		1 (0.5%)	0 (0.0%)	
– Unknown	144 (58.8%)	182 (57.6%)		130 (59.9%)	144 (66.4%)	
Fracture at OP entry, n (%)	143 (58.4%)	204 (64.6%)	0.13	127 (58.5%)	137 (63.1%)	0.10

**Table 3:** Characteristics of patients with a hip fracture at OP registry entry.

Characteristic	Zol (N=33)	Dmab (N=51)	SMD
Gender			0.2
– Female	17 (52%)	31 (61%)	
– Male	16 (48%)	20 (39%)	
Age at treatment	77 ± 7	81 ± 7	0.6
BMI	30.2 ± 5.1	30.4 ± 6.3	0.035
CVD event	16 (48%)	26 (51%)	0.050
Stroke event	6 (18%)	8 (16%)	0.067
HbA1c	6.57 ± 0.71	6.72 ± 0.73	0.2
Alcohol use	0 (0%)	0 (0%)	–
eGFR category			0.8
– (60+)	16 (48%)	20 (39%)	
– (0–30]	0 (0%)	7 (14%)	
– (30–60]	5 (15%)	15 (29%)	
– Missing	12 (36%)	9 (18%)	
eGFR	73 ± 18	59 ± 26	0.7
– Unknown	12	9	
Statin users	24 (73%)	47 (92%)	0.5
Diabetes duration	7.9 ± 6.0	9.4 ± 6.6	0.2
Microvascular events	19 (58%)	33 (65%)	0.3
CCI	4 ± 4	4 ± 3	0.15
Smoking status			
– Current smoker	5 (15%)	5 (9.8%)	
– Never	27 (82%)	46 (90%)	
– Past smoker	1 (3.0%)	0 (0%)	

Zol, zoledronic acid; Dmab, denosumab; SMD, standardized mean difference; SD, standard deviation; HbA1c, glycated hemoglobin; eGFR, estimated glomerular filtration rate; CCI, Charlson comorbidity index; OP, osteoporosis.

**Figure 3:**
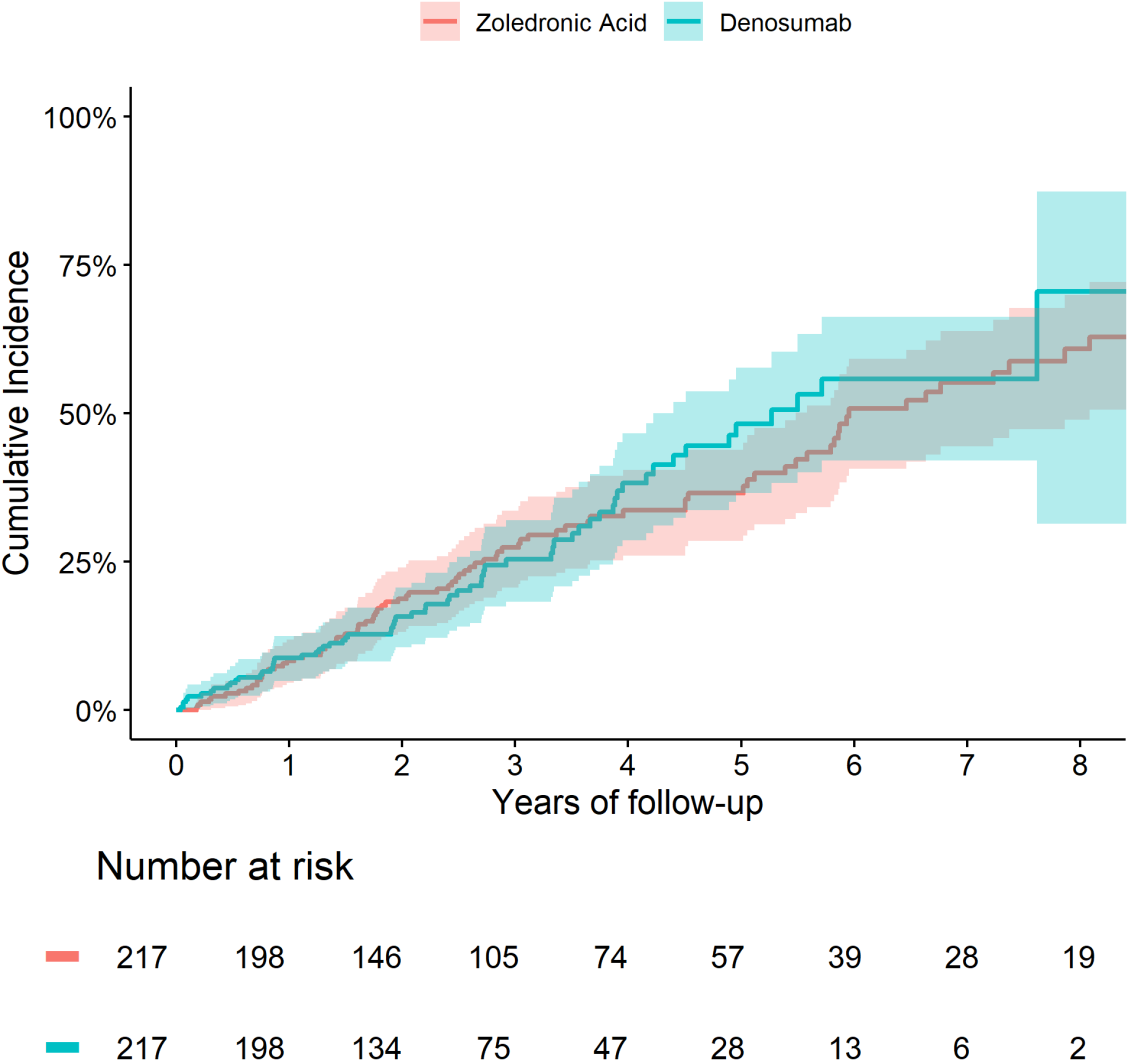
Cumulative incidence of death in subjects older than 75 treated with Zol *vs* Dmab.

**Figure 4:**
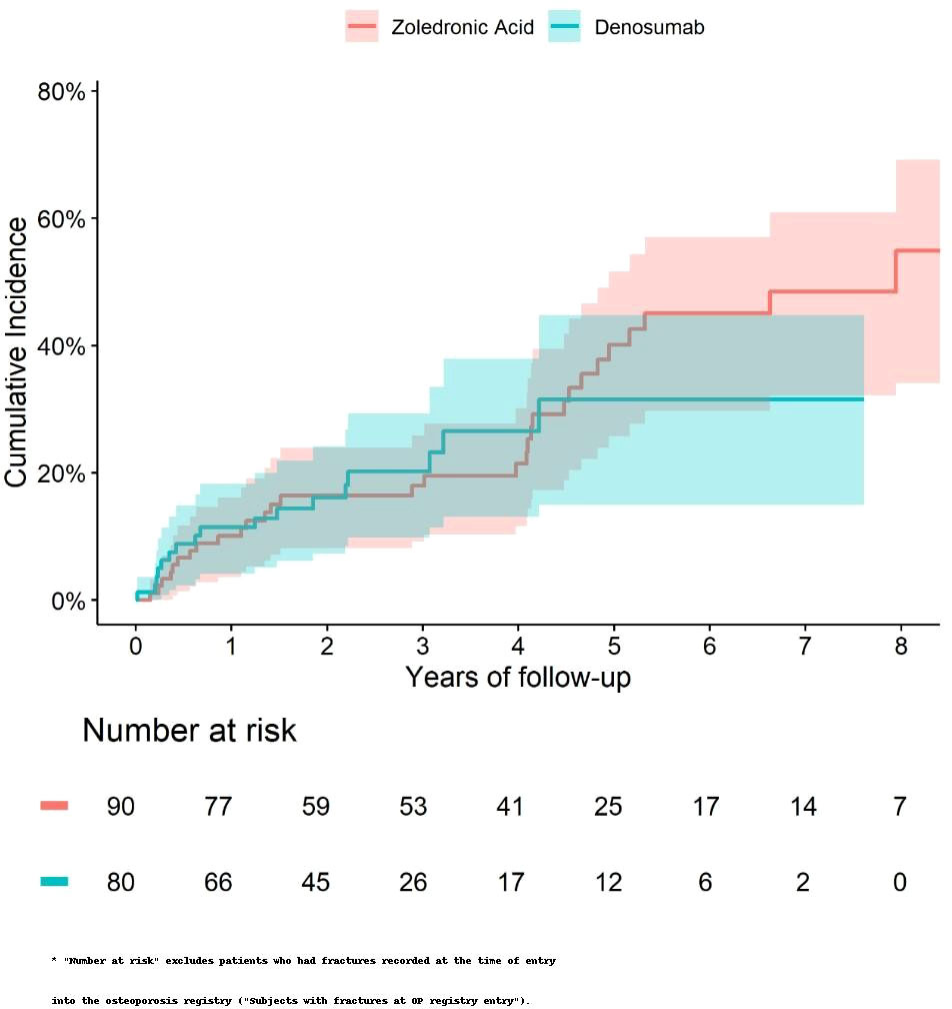
Cumulative incidence of fractures in subjects older than 75 and fracture-free at OP registry entry treated with Zol *vs* Dmab.

The cumulative incidence of death and fractures were similar between the two treatment groups (**Figures 3**, **4**). We did not observe any significant differences in the risk of fracture HR=0.90 (0.49 to 1.65) or death HR= 1.04 (0.74 to 1.45) between Zol and Dmab treatments in patients above the age of 75.

In this propensity score–matched cohort, a total of 84 hip fractures were recorded at osteoporosis registry entry, 51 were then treated with Dmab and 33 with Zol. The mean age at the time of fracture was 80.9 years (± 6.8), the mean body mass index (BMI) was 30.5 kg/m² (± 6.1), and the mean estimated glomerular filtration rate (eGFR) was 63.6 mL/min/1.73 m² (± 19.4); 60.4% of the fractures occurred in women. Their baseline characteristics are summarized in **Table 3**.

**Figure 5:**
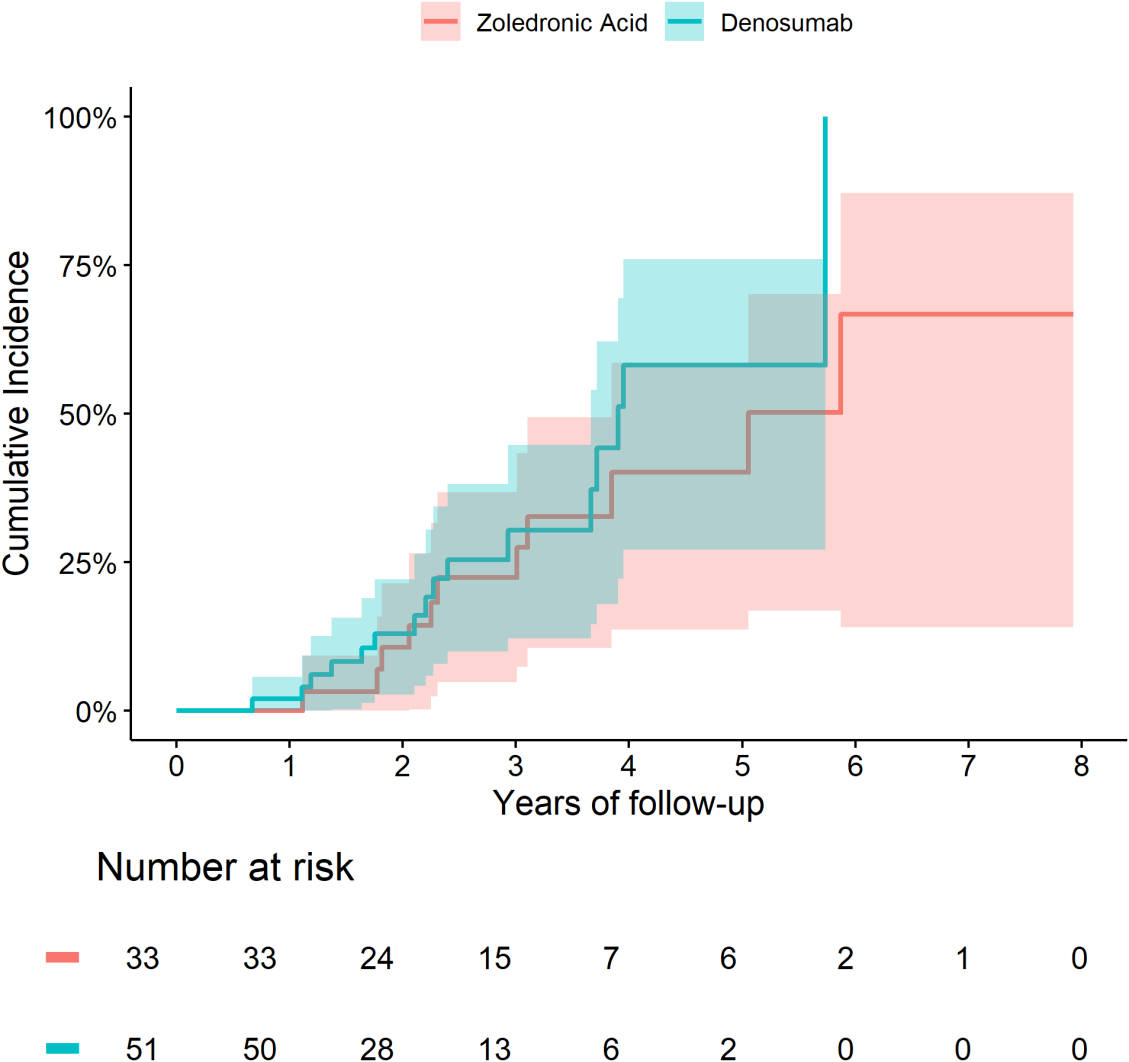
Cumulative incidence of death among patients with a hip fracture at index treated with Zol *vs* Dmab.

Patients treated with denosumab (Dmab) were significantly older, had lower estimated glomerular filtration rates (eGFR), and used statins more frequently compared to those treated with zoledronic acid (Zol). The cumulative incidence of death was similar between the two groups (**Figure 5**). In a multivariate analysis adjusting for age, eGFR the risk of death remained comparable (hazard ratio [HR] = 0.92; 95% confidence interval [CI]: 0.37–2.29).

## 4 Discussion

In this population-based cohort study, Zoledronic Acid and Denosumab were associated with similar fracture and death rates in patients with concurrent osteoporosis and type 2 diabetes. The findings were consistent among subjects older than 75 years and subjects after a hip fracture, although caution is needed in interpreting underpowered subgroup analyses.

Our findings suggest comparable effectiveness between two widely prescribed anti-resorptive agents. These results are consistent with prior observational studies in the general osteoporotic population (19–21). This suggests that the efficacy of both zoledronic acid and denosumab may extend across diverse patient populations, including those with elevated fracture risk due to comorbidities such as type 2 diabetes.

While the study by Curtis et al. (2023) demonstrated greater efficacy of denosumab over zoledronic acid in the general osteoporotic population, our findings suggest both agents may offer comparable effectiveness. This discrepancy may reflect differences in population characteristics, such as the presence of diabetes-related bone alterations, renal function, and overall comorbidity burden, which may influence treatment response in this high-risk group.

The strengths of this study include a large cohort of patients, which is representative of the Israeli population as MHS is the second largest healthcare provider and insurer in Israel covering approximately three million people with a countrywide distribution. It provides a good quality real-world data on multiple relevant variables, including BMD results, and adjudicated fracture diagnoses. Our cohort represents patients from a real-life setting: older subjects, with lower kidney function and substantial cardiovascular risk factors, subjects which are usually less represented in randomized controlled trials. Our study reflects the general practice in Israel and provides reassurance of the safety and efficacy of two widely prescribed antiresorptive agents in an especially high-risk population. We performed a direct comparison of biannual subcutaneous denosumab initiators versus annual intravenous zoledronic acid initiators and had a relatively long follow up.

Our study has several limitations. First, even though we controlled for many potential confounders using a propensity score matching method, there is a potential for unmeasured or residual confounding. Second, the duration of treatment was not uniformly recorded and could not be reliably assessed; therefore, comparisons were based on treatment initiation rather than cumulative exposure. The data on some covariates was incomplete, including BMI, smoking status and GFR, as well as calcium and vitamin D supplement exposure, as those are mainly purchased over the counter. Third, our study may not have adequate power to detect a significant difference between the treatments for some of the individual endpoints and subgroup analyses. Fourth, this study was not designed to examine other potential safety events such as atypical fractures, hypocalcemia and osteonecrosis of the jaw.

One interesting observation was the relatively low HbA1C levels in our study population. The diabetes registry may include patients with pre-diabetes which may have led to lower mean HbA1C levels in our cohort. On the other hand, these values are extracted from real-world data of an elderly population with type 2 diabetes and osteoporosis, with relatively low BMI, and therefore may reflect a milder disease course in this population. In the *post hoc* analysis of the 3-year, placebo-controlled FREEDOM study and 7-year extension, which included postmenopausal women with osteoporosis and diabetes, HbA1c levels were not reported, however, mean fasting glucose levels were 150.5 (54.2) and 147 (44.5) in placebo versus denosumab-treated patients respectively (17). These levels are equivalent to an HbA1c of approximately 6.7% (34) which is similar to the values reported in our study. We aimed to evaluate the validity of our results in diabetic patients with a poorer glycemic control, but the number of patients was too modest and should therefore be the focus of a future study.

Finally, in the subgroup of older participants, we observed a numerically lower risk of fracture and mortality compared to the overall cohort. However, these differences were not statistically significant and likely reflect the limited sample size in this subgroup, which reduces statistical power. Therefore, we caution against overinterpretation of these findings. The result may reflect selection bias, as older adults offered treatment may have been healthier or more adherent than those excluded. These exploratory findings highlight the need for larger, adequately powered studies to assess treatment outcomes in very old individuals with osteoporosis and type 2 diabetes.

## 5 Conclusions

Our findings suggest the equivalent efficacy of Zoledronic Acid and Denosumab in managing osteoporotic fractures and mortality among patients with type 2 diabetes, offering valuable insight into treatment strategies in this vulnerable population. Further study is needed to establish the efficacy and safety of different therapeutic options in diabetic subjects especially in very old individuals and those with poor glycemic control.

## Data Availability

The raw data supporting the conclusions of this article will be made available by the authors, without undue reservation.

## References

[1] Clynes MA Harvey NC Curtis EM Fuggle NR Dennison EM Cooper C. The epidemiology of osteoporosis. Br Med Bull. (2020) 133:105–17. doi: 10.1093/bmb/ldaa005, PMID: 32282039 PMC7115830

[2] NIH Consensus Development Panel on Osteoporosis Prevention, Diagnosis, and Therapy. Osteoporosis prevention, diagnosis, and therapy. JAMA. (2001) 285:785–95. doi: 10.1001/jama.285.6.785, PMID: 11176917

[3] Johnston CB Dagar M. Osteoporosis in older adults. Med Clin North Am. (2020) 104:873–84. doi: 10.1016/j.mcna.2020.06.004, PMID: 32773051

[4] Hofbauer LC Busse B Eastell R Ferrari S Frost M Müller R. Bone fragility in diabetes: novel concepts and clinical implications. Lancet Diabetes Endocrinol. (2022) 10:207–20. doi: 10.1016/S2213-8587(21)00347-8, PMID: 35101185

[5] Hsu JY Cheng CY Hsu CY. Type 2 diabetes mellitus severity correlates with risk of hip fracture in patients with osteoporosis. Neth J Med. (2018) 76:65–71., PMID: 29515003

[6] Lui DTW Lee CH Chan YH Chow WS Fong CHY Siu DCW. HbA1c variability in addition to mean HbA1c predicts incident hip fractures in Chinese people with type 2 diabetes. Osteoporos Int. (2020) 31:1955–64. doi: 10.1007/s00198-020-05395-z, PMID: 32385660

[7] Li CI Liu CS Lin WY Meng NH Chen CC Yang SY. Glycated hemoglobin level and risk of hip fracture in older people with type 2 diabetes: a competing risk analysis of Taiwan diabetes cohort study. J Bone Miner Res. (2015) 30:1338–46. doi: 10.1002/jbmr.2462, PMID: 25598134

[8] Schwartz AV. Efficacy of osteoporosis therapies in diabetic patients. Calcif Tissue Int. (2017) 100:165–73. doi: 10.1007/s00223-016-0177-8, PMID: 27461216

[9] Farr JN Drake MT Amin S Melton LJ McCready LK Khosla S. *In vivo* assessment of bone quality in postmenopausal women with type 2 diabetes. J Bone Miner Res. (2014) 29:787–95. doi: 10.1002/jbmr.2106, PMID: 24123088 PMC3961509

[10] Samelson EJ Demissie S Cupples LA Atkinson EJ Broe KE Hannan MT. Diabetes and deficits in cortical bone density, microarchitecture, and bone size: the Framingham HR-pQCT study. J Bone Miner Res. (2018) 33:54–62. doi: 10.1002/jbmr.3240, PMID: 28929525 PMC5771832

[11] Patsch JM Burghardt AJ Yap SP Soung D Link TM Majumdar S. Increased cortical porosity in type 2 diabetic postmenopausal women with fragility fractures. J Bone Miner Res. (2013) 28:313–24. doi: 10.1002/jbmr.1763, PMID: 22991256 PMC3534818

[12] Eastell R Vittinghoff E Lui LY Cauley JA Gao L Danielson ME. Diabetes mellitus and the benefit of antiresorptive therapy on fracture risk. J Bone Miner Res. (2022) 37:2121–31. doi: 10.1002/jbmr.4697, PMID: 36065588 PMC10092457

[13] Vilaca T Schini M Harnan S Welch V Bevan G Rondón M. The risk of hip and non-vertebral fractures in type 1 and type 2 diabetes: a systematic review and meta-analysis update. Bone. (2020) 137:115457. doi: 10.1016/j.bone.2020.115457, PMID: 32480023

[14] Vestergaard P. Discrepancies in bone mineral density and fracture risk in patients with type 1 and type 2 diabetes—a meta-analysis. Osteoporos Int. (2007) 18:427–44. doi: 10.1007/s00198-006-0253-4, PMID: 17068657

[15] Hygum K Starup-Linde J Harslof T Vestergaard P Langdahl BL Bagger Y. Mechanisms in endocrinology: diabetes mellitus, a state of low bone turnover—a systematic review and meta-analysis. Eur J Endocrinol. (2017) 176:R137–57. doi: 10.1530/EJE-16-0652, PMID: 28049653

[16] Keegan TH Schwartz AV Bauer DC Sellmeyer DE de Papp AE Strotmeyer ES. Effect of alendronate on bone mineral density and biochemical markers of bone turnover in type 2 diabetic women: the Fracture Intervention Trial. Diabetes Care. (2004) 27:1547–53. doi: 10.2337/diacare.27.7.1547, PMID: 15220226

[17] Ferrari S Eastell R Napoli N Cummings SR Cauley JA Lang TF. Denosumab in postmenopausal women with osteoporosis and diabetes: subgroup analysis of FREEDOM and FREEDOM Extension. Bone. (2020) 134:115268. doi: 10.1016/j.bone.2020.115268, PMID: 32058020

[18] Beaudoin C Jean S Bessette L Josse RG Adachi JD Papaioannou A. Denosumab compared to other treatments to prevent or treat osteoporosis in individuals at risk of fracture: a systematic review and meta-analysis. Osteoporos Int. (2016) 27:2835–44. doi: 10.1007/s00198-016-3607-6, PMID: 27120345

[19] Choi NK Solomon DH Tsacogianis TN Landon JE Song HJ Kim SC. Comparative safety and effectiveness of denosumab versus zoledronic acid in patients with osteoporosis: a cohort study. J Bone Miner Res. (2017) 32:611–7. doi: 10.1002/jbmr.3019, PMID: 27736041 PMC5340628

[20] Curtis JR Arora T Liu Y Chatterjee A Varun V Saag KG. Comparative effectiveness of denosumab versus zoledronic acid among postmenopausal women with osteoporosis in the U.S. Medicare program. Vancouver Convention Centre, Vancouver, BC, Canada: American Society for Bone and Mineral Research Annual Meeting (2023).

[21] Alarkawi D Tran T Chen W Zhang X Patel R Singh G. Denosumab and mortality in a real-world setting: a comparative study. J Bone Miner Res. (2023) 38:1757–70. doi: 10.1002/jbmr.4930, PMID: 37915252

[22] Huang HK Chuang AT Liao TC Shao SC Liu PP Tu YK. Denosumab and the risk of diabetes in patients treated for osteoporosis. JAMA Netw Open. (2024) 7:e2354734. doi: 10.1001/jamanetworkopen.2023.54734, PMID: 38335002 PMC10858399

[23] Lyu H Zhao SS Zhang L Cai X Li Y Wang J. Denosumab and incidence of type 2 diabetes among adults with osteoporosis: population-based cohort study. BMJ. (2023) 381:e073435. doi: 10.1136/bmj-2022-073435, PMID: 37072150 PMC10111187

[24] Bonnet N Bourgoin L Biver E Douni E Ferrari S Buchonnet G. RANKL inhibition improves muscle strength and insulin sensitivity and restores bone mass. J Clin Invest. (2019) 129:3214–23. doi: 10.1172/JCI125915, PMID: 31120440 PMC6668701

[25] Rabenda V Mertens R Fabri V Vanoverloop J Vannecke C De Groote S. Adherence to bisphosphonates therapy and hip fracture risk in osteoporotic women. Osteoporos Int. (2008) 19:811–8. doi: 10.1007/s00198-007-0493-6, PMID: 17999022

[26] de Groen PC Lubbe DF Hirsch LJ Daifotis AG Stephenson W Freedholm D. Esophagitis associated with the use of alendronate. N Engl J Med. (1996) 335:1016–21. doi: 10.1056/NEJM199610033351402, PMID: 8793925

[27] Cummings SR San Martin J McClung MR Siris ES Eastell R Peterson MC. Denosumab for prevention of fractures in postmenopausal women with osteoporosis. N Engl J Med. (2009) 361:756–65. doi: 10.1056/NEJMoa0809493, PMID: 19671655

[28] Black DM Delmas PD Eastell R Cummings SR Harris ST Funke ME. Once-yearly zoledronic acid for treatment of postmenopausal osteoporosis. N Engl J Med. (2007) 356:1809–22. doi: 10.1056/NEJMoa067312, PMID: 17476007

[29] McClung MR Lewiecki EM Cohen SB Bolognese MA Wooddell CM Patt J. Denosumab in postmenopausal women with low bone mineral density. J Clin Endocrinol Metab. (2006) 91:3941–7. doi: 10.1210/jc.2006-0610, PMID: 16968791

[30] Chodick G Heymann AD Shalev V Kokia E Ross S Lavie O. The epidemiology of diabetes in a large Israeli HMO. Eur J Epidemiol. (2003) 18:1143–6. doi: 10.1023/b:ejep.0000006635.36802.c8, PMID: 14758871

[31] Shalev V Chodick G Goren I Silber H Kokia E Heymann AD. The use of an automated patient registry to manage and monitor cardiovascular conditions and related outcomes in a large health organization. Int J Cardiol. (2011) 152:345–9. doi: 10.1016/j.ijcard.2010.08.002, PMID: 20826019

[32] Goldshtein I Chandler J Shalev V Heymann AD Kookia E Berkowitz O. Osteoporosis in the community: findings from a novel computerized registry in a large health organization in Israel. J Aging Res Clin Pract. (2015) 4:59–65. doi: 10.14283/jarcp.2015.43

[33] Kanis JA Johnell O Oden A Johansson H McCloskey E Glüer CC. FRAX and the assessment of fracture probability in men and women from the UK. Osteoporos Int. (2008) 19:385–97. doi: 10.1007/s00198-007-0543-5, PMID: 18292978 PMC2267485

[34] Nathan DM Kuenen J Borg R Zheng H Schoenfeld D Heine RJ. Translating the A1C assay into estimated average glucose values. Diabetes Care. (2008) 31:1473–8. doi: 10.2337/dc08-0545, PMID: 18540046 PMC2742903

